# Spontaneous Pneumomediastinum in a Healthy Young Male: A Case Report from Riyadh, Saudi Arabia

**DOI:** 10.7759/cureus.4442

**Published:** 2019-04-12

**Authors:** Mohammed Alnamlah, Louay S Abdulkarim, Lama AlFakhri, Abdulaziz Alali

**Affiliations:** 1 Emergency Medicine, College of Medicine at Alfaisal University, Riyadh, SAU; 2 Miscellaneous, College of Medicine at Alfaisal University, Riyadh, SAU; 3 Emergency Medicine, King Faisal Specialist Hospital and Research Center, Riyadh, SAU

**Keywords:** spontaneous pneumomediastinum, hamman’s sign, chest pain, saudi arabia

## Abstract

Pneumomediastinum is defined as the presence of air in the mediastinum. Trauma to the nearby organs can cause air to escape into surrounding tissues that may manifest clinically as severe chest pain, voice change, or shortness of breath. However, pneumomediastinum can present spontaneously in healthy individuals with no inciting factors in which case the condition is termed spontaneous pneumomediastinum (SPM). Pneumomediastinum can be challenging to manage due to the absence of clear guidelines for the diagnosis and management.

We present the case of a 21-year-old with no previous medical history who presented with chest pain that was aggravated by speech and breath. The pain was of sudden onset preceded by smoking at 2:00 am. The patient was tachycardic, tachypnoeic with crepitation on palpation and a crunch sound (Hamman’s sign) on auscultation. The patient rated the pain as 5/10 on a 11-point numerical pain rating scale, which then evolved to 10/10. The patient did not have fever, loss of consciousness (LOC), diaphoresis, history of trauma, or previous similar presentation. There were no other associated symptoms. A chest X-ray (posteroanterior (PA) and lateral view) showed pneumomediastinum, but laboratory tests results were otherwise normal. The patient was observed in the emergency room overnight. He remained stable, his tachycardia settled, and there was no leukocytosis or desaturation; however, tachypnea was observed. His pain symptoms were treated with analgesia as needed and the patient was discharged home in a stable condition, to be followed on an outpatient basis.

Spontaneous pneumomediastinum can be challenging to manage due to the lack of reliable incidence data as well as the absence of clear management guidelines. Further research will aid in understanding the true incidence of SPM in Saudi Arabia and help in establishing a consensual approach and treatment guidelines to deal with SPM in otherwise healthy individuals. To the best of our knowledge, this is the first case of SPM in a young male reported from a tertiary hospital in Riyadh, Saudi Arabia.

## Introduction

Pneumomediastinum is defined as the presence of air in the mediastinum. The mediastinum is flanked by the lungs and contains most of the thoracic organs. Trauma to the nearby organs might cause air to escape into surrounding tissues, which may manifest clinically as severe chest pain, voice change or shortness of breath. On auscultation, it is recognized as crunching sounds that parallel the cardiac cycle (Hamman’s crunch). When pneumomediastinum presents in healthy individuals with no obvious inciting factor, it is called spontaneous pneumomediastinum (SPM) [1].

Spontaneous pneumomediastinum is a rare disease that most commonly affects young males secondary to alveolar rupture; it can also be associated with asthma, especially in children. It has a benign course and usually resolves spontaneously, which can be detected by mediastinal gas resolution using a chest X-ray [2]. The incidence of pneumomediastinum is thought to be 1/25,000, occurring in people aged 5-34 years with the majority of patients being males, who account for 76% of cases [1]. SPM can be challenging to manage due to the lack of reliable incidence data as well as the absence of clear management guidelines. Thus, the management of SPM varies from one center to another. To the best of our knowledge, this is the first case of SPM in a young male reported from a tertiary hospital in Riyadh, Saudi Arabia, and we hope that this case will aid in understanding the true incidence of SPM in Saudi Arabia and help in establishing a consensual approach and treatment guidelines.

## Case presentation

A 21-year-old Saudi male with no previous medical history presented to the emergency department with chest pain that was stabbing in nature. The chest pain was located in the central and left regions of the chest with no radiation. The pain was aggravated by speech and breathing and there were no relieving factors. The pain was of sudden onset preceded by smoking at 2:00 am. The patient rated the pain as 5/10 on a 11-point numerical pain rating scale, which then evolved to 10/10. The patient did not have fever, shortness of breath (SOB), loss of consciousness (LOC), diaphoresis, trauma or previous similar presentation. There were no other associated symptoms.

On examination, the patient was alert and oriented with no acute distress. His vital signs revealed a blood pressure of 104/64 mmHg, heart rate of 105/min, respiratory rate of 20/min, temperature of 36.9C, and he was saturating 100% on room air. A respiratory examination showed tachypnea. The patient had crepitation on palpation and a crunch sound (Hamman’s sign) was heard on auscultation. A cardiovascular examination was unremarkable except for tachycardia. A gastrointestinal examination showed a soft, non-tender and non-distended abdomen. His musculoskeletal examination results were within normal limits and the integumentary system had no acute disease. His neurologic examination was unremarkable.

**Figure 1:**
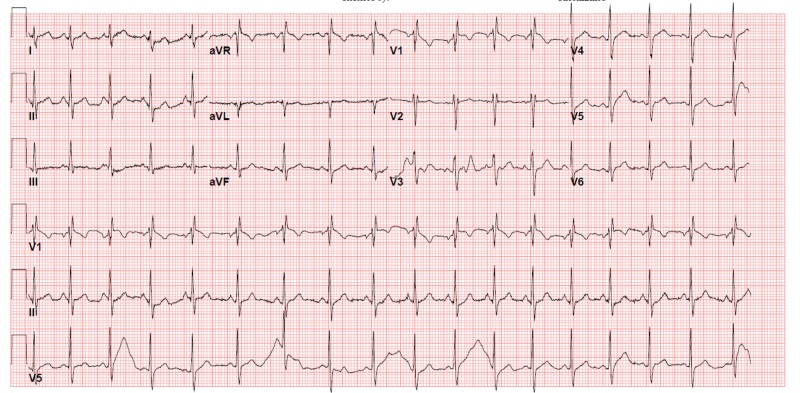
Electrocardiogram (ECG) showing sinus tachycardia

There were no significant findings in his lab results, as he had a white cell count of 12.13 count/L, a hemoglobin of 166 g/L, and a platelet count of 168 count/L. His cardiac enzymes showed CK 239 U/L and troponin T 0.007 Ug/L. His liver function tests, kidney function tests, venous blood gas result, and anion gap were all within the normal range. An electrocardiogram (ECG) was ordered upon presentation to the emergency department (ED) (Figure 1).

**Figure 2:**
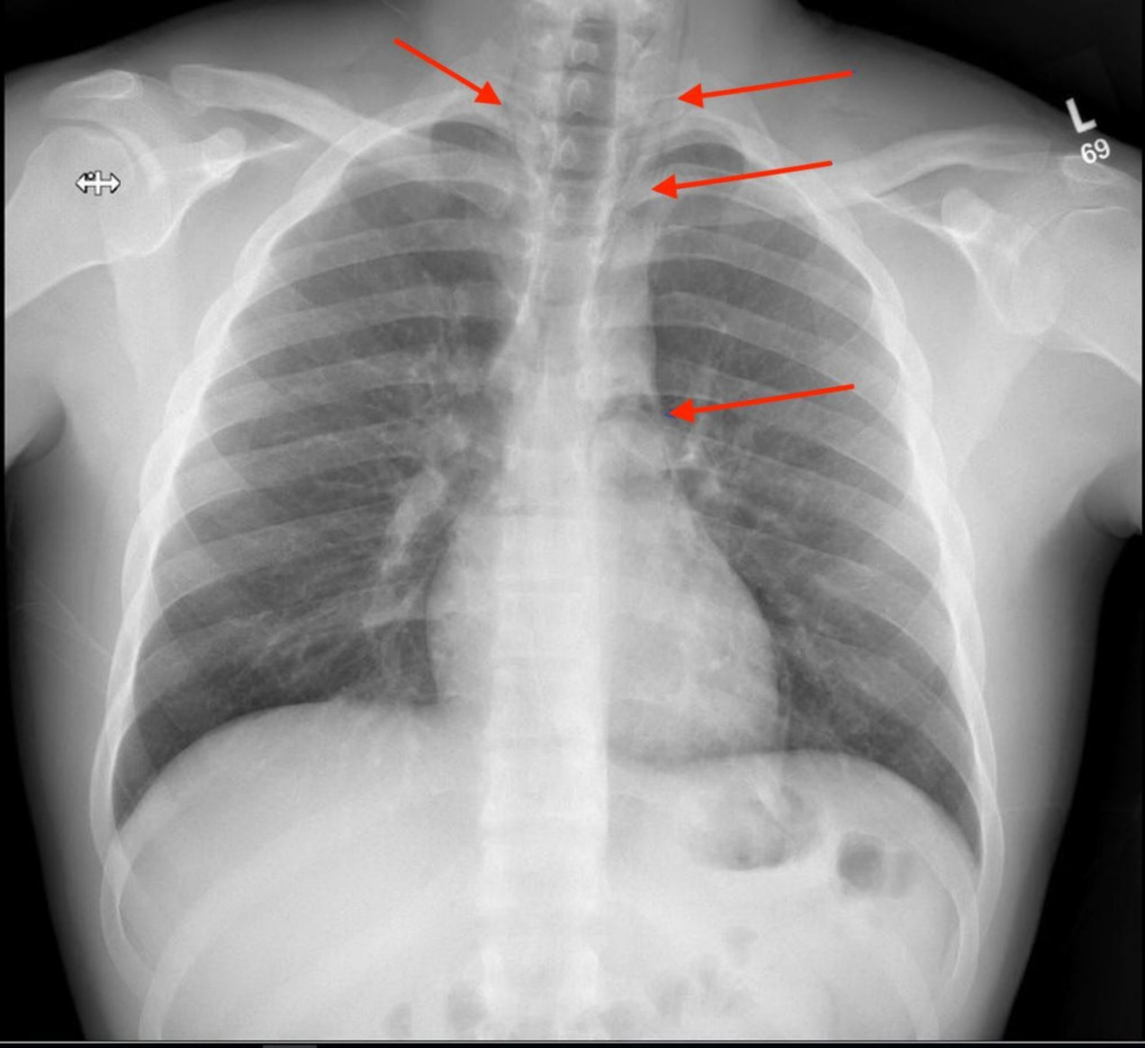
Linear lucencies were noted in the middle and superior mediastinum indicating the possibility of underlying pneumomediastinum

**Figure 3:**
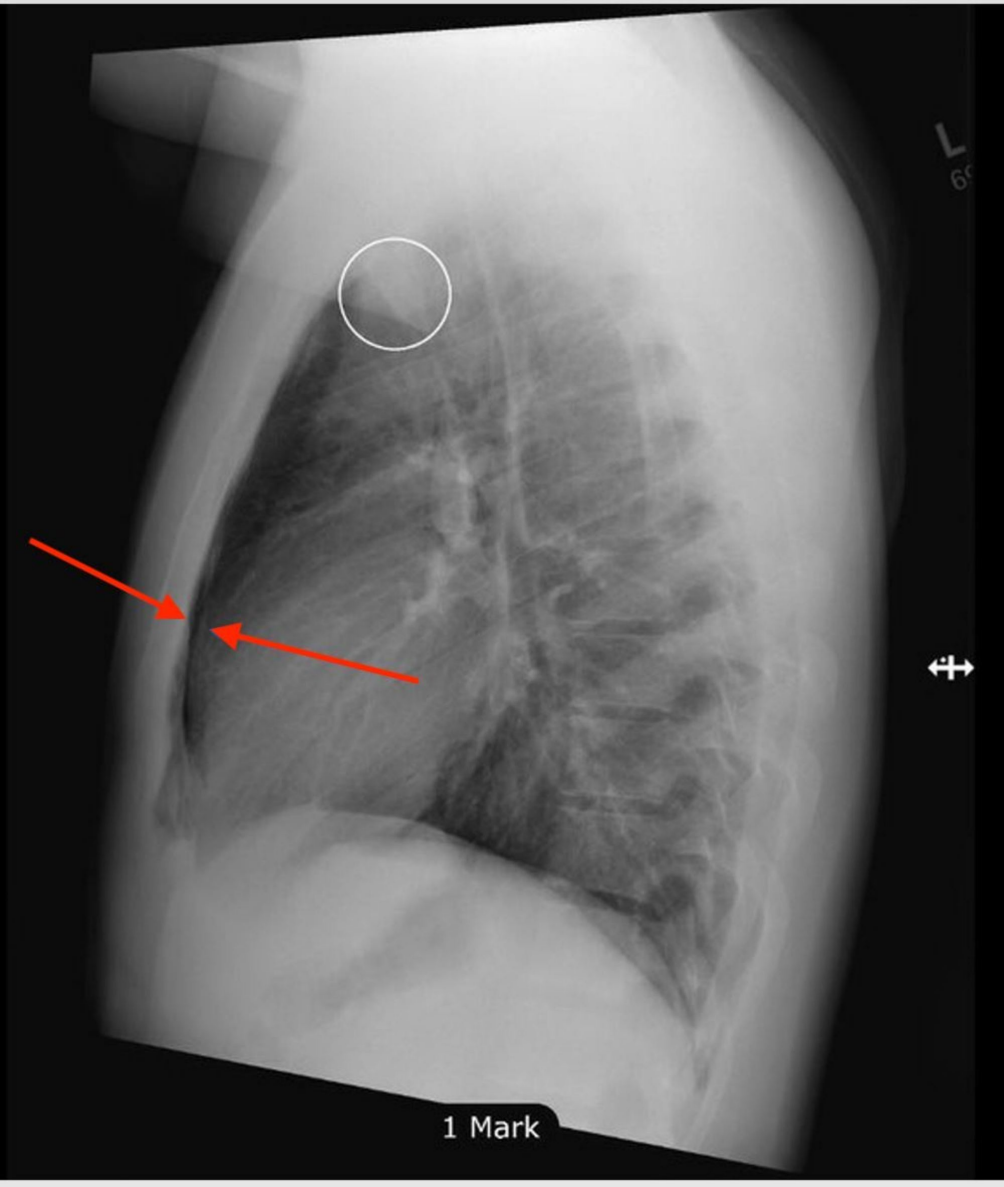
Linear lucencies were noted in the middle and superior mediastinum indicating the possibility of underlying pneumomediastinum

Posteroanterior and lateral chest X-rays were obtained to confirm the diagnosis and pneumomediastinum was identified (Figures 2-3).

The patient was kept overnight for observation, with repeat labs ordered the following day. He had a white blood count of 8.58 count/L, hemoglobin of 152 g/L, and a platelet count of 154 count/L. The patient was discharged home with acetaminophen for pain. A follow-up visit after three weeks and a CT chest with contrast before follow-up was planned. However, the patient was lost to follow-up.

## Discussion

Hamman’s syndrome or spontaneous pneumomediastinum (SPM) was first reported in 1939 by Louis Hamman. SPM is defined as the presence of air in the mediastinum without any precipitating factors or triggering etiologies [3].

The exact incidence of SPM has not been clearly identified because the only published reports available in the literature are case reports and small case series. To the best of our knowledge, this is the first case of SPM in a young male reported from a tertiary hospital in Riyadh, Saudi Arabia.

The presence of air in the mediastinum is due to the pressure difference that occurs as a consequent event to alveolar rupture. Coughing, vomiting, intense exertion, and Valsalva maneuvers can cause a sudden increase in the intrathoracic pressure leading to the pathophysiology of the disease. Most patients present with the classic triad that includes chest pain, dyspnea, and subcutaneous emphysema of the neck [4].

A retrospective study that was conducted in Barcelona between 1990 and 2006 reported that 85% of the patients diagnosed with SPM presented with centrally located chest pain and that the time between the onset of symptoms and the hospital visit ranged between 5-72 hours [3]. Similarly, our patient presented with an acute central chest pain as his chief complaint. The pain started at 2:00 am, and he presented to our emergency department around 7:00 am.

Another study that was conducted by Dajer-Fadel, who used a Medline search between 1990 and 2012, revealed that the most common signs and symptoms patients usually present with are pain, dyspnea, subcutaneous emphysema, and Hamman’s sign [5].

In recent studies, Hamman’s sign was identified in 30% of cases [6]. The established auscultative sign of SPM is heard with each beat of the heart against the air filled tissues. The sound is heard as crackles or crunch sounds, which is similar to the auscultation finding in our patient during the respiratory examination.

Following the pattern mentioned in most of the literature, our patient’s laboratory results had no specific abnormalities. Nonetheless, one retrospective study had either leukocytosis or increased C-reactive protein (CRP) in 80% of their patients [7].

It stands to reason that the most important diagnostic test to confirm SPM is radiography. Thus in our case, posteroanterior and lateral chest radiographs were done to confirm the diagnosis. A lateral view is recommended in such cases, as 50% of all cases might remain undiagnosed if only a posteroanterior radiograph is taken [3]. According to the literature, the diagnosis of SPM is established with a clinical examination and a simple chest X-ray. However, if the diagnosis of SPM was skeptical or when there is a suspension of a rupture of the hollow viscera, a CT scan should be added to aid the diagnosis along with other tests [8]. Subsequently, we booked a CT study for our patient in his follow-up appointment in case his symptoms did not improve, but he was lost to follow-up.

When considering spontaneous pneumomediastinum one should keep in mind various differential diagnoses. One of the most important and possibly dangerous differentials include Boerhaave’s syndrome. It is defined as spontaneous esophageal rupture that usually occurs after forceful emesis [9]. Since SPM and Boerhaave’s syndrome can present with similar clinical features, it is important to rule it out as soon as possible. In such cases, bronchoscopy is the definitive test; CT scan or contrast-enhanced swallow may be used as well [3]. Diagnosing this entity early and referring the patient for surgery has a great impact on the morbidity and mortality [9]. Other differentials that are less potentially dangerous yet should be considered when suspecting SPM include musculoskeletal disorders, pericarditis, acute coronary syndrome, and pulmonary embolism [3].

Conservative management has proven to be the mainstay of treatment in SPM, which includes bed rest, analgesia, and close observation. A retrospective study that was conducted for seven years in Japan concluded that patients may recover from SPM without admission or the need for prophylactic antibiotics [10]. As mentioned in the literature, the clinical time course in which the symptoms improve vary between 24 hours to 48 hours and the complete X-ray resolution generally takes about a week [6]. A study by Mondello in 2007 evaluated 336 cases and out of all the cases, only two cases were identified with secondary pneumothorax as a complication of SPM [6]. Having said that, the complications associated with spontaneous pneumomediastinum are minimum and rarely occur. Therefore, long time follow-up is not recommended as spontaneous pneumomediastinum is a benign entity with a spontaneous recovery, in addition to the fact that there is a low incidence of recurrence reported in the literature.

## Conclusions

Spontaneous pneumomediastinum has the tendency to resolve spontaneously without the need of invasive treatment and investigation methods. Thus, it can be challenging to manage due to the absence of clear guidelines for the diagnosis and management. In addition, there is little or no data on the incidence of SPM in the region. Therefore, we presented this as the first case to be reported in a tertiary hospital in Riyadh, Saudi Arabia, in the hopes that it will aid in establishing both the incidence and the management guidelines for treating SPM in Saudi Arabia. In order for that to happen, more cases need to be documented and more than one center needs to be involved on a national and international level.
